# Bongkrekic acid facilitates glycolysis in cultured cells and induces cell death under low glucose conditions

**DOI:** 10.1016/j.bbrep.2019.100683

**Published:** 2019-08-29

**Authors:** Arihiro Kano, Takuma Iwasaki, Mitsuru Shindo

**Affiliations:** a Institute for Materials Chemistry and Engineering, Kyushu University, 6-1 Kasuga-koen, Kasuga, Fukuoka, 819-0395, Japan; b Interdisciplinary Graduate School of Engineering Sciences, Kyushu University, 6-1 Kasuga-koen, Kasuga, Fukuoka, 819-0395, Japan

**Keywords:** Bongkrekic acid, Mitochondria, Glycolysis, Tumor cells, Warburg effect, ANT, adenine nucleotide translocator, BKA, bongkrekic acid, MPTP, mitochondrial permeability transition pores, OXPHOS, oxidative phosphorylation, PBS, phosphate-buffered saline

## Abstract

Bongkrekic acid (BKA) inhibits adenine nucleotide translocator (ANT) and suppresses ADP/ATP exchange in the mitochondrial inner membrane. Previously, we demonstrated that BKA exhibited cytotoxic effects on 4T1 tumor cells, depending on the cell number in the culture, but not on NIH3T3 cells. However, the cause of this differential sensitivity was unelucidated. Here we demonstrate that BKA reduced the O_2_ consumption in both cell lines and increased the mitochondrial membrane potential, thereby facilitating glucose consumption. BKA reduced cellular ATP in 4T1 cells in a dose-dependent manner but not in NIH3T3 cells. The cellular ATP of 4T1 cells was decreased with a reduced glucose concentration in the media, but that of NIH3T3 cells remained constant. We also demonstrated that BKA-induced cell death in both cell lines in low glucose media; however, the susceptibility to the reduced glucose concentration was slightly higher in 4T1 cells, which may be attributed to the difference in the dependency on glycolysis as their energy source. These results indicate that 4T1 tumor cells rely heavily on glucose for energy production. Our data demonstrate that BKA disturbs ATP production in mitochondria and increases the susceptibility to a low glucose condition.

## Highlights

• Bongkrekic acid decreases cellular ATP in cancer cells.
• Bongkrekic acid decreases mitochondrial OXPHOS and enhances glycolysis in cells.
• Bongkrekic acid induces cell death under low glucose conditions.

## 1 Introduction

Bongkrekic acid (BKA) is one of the toxic compounds produced by the bacterium *Burkholderia gladioli* pathovar *cocovenenans* and was first isolated by van Veen and Mertens [1]. BKA is as an inhibitor of adenine nucleotide translocator (ANT), also known as ADP/ATP translocase and ADP/ATP carrier, in the mitochondrial inner membrane [2]. ANT supplies ATP from the mitochondria to the cytosol in exchange for cytosolic ADP. The mechanisms of ANT for ADP/ATP exchange has been well documented in others [[3], [4], [5]]. The ANT genes express four isoforms in humans (ANT1–4) and three isoforms in mice (ANT3 is not present). The ANT1 gene is predominantly expressed in heart and muscle, and the ANT2 gene is mainly expressed in lymphoid cells and the liver. The ANT3 gene is expressed abundantly but at a low level, and ANT4 is only expressed in brain, liver, and testis [6,7]. Interestingly, it has been reported that the ANT2 gene is abundantly expressed in cancer cells and aids in the importation of ATP produced by glycolysis from the cytosol to the mitochondria [8]. The glycolytic ATP in mitochondria is hydrolyzed by F1F0-ATP synthase in a reverse reaction that produces a proton motive force, which maintains the mitochondrial membrane potential. However, it has recently been reported that ATP importation is independent of ANT in HepG2 and A549 cells [9]. Thus, the role of ANT2 in cancer cells remains controversial.

Although BKA was first isolated as a cause of the poisoning of the fermented food, the physiological and cell biological understanding has not been sufficiently achieved yet [10]. We have previously demonstrated that BKA reduces the viability of 4T1 murine mammary cancer cells but not of NIH3T3 immortalized fibroblasts [11]. The cytotoxic effects of BKA depend on the number of seeded cells and are attenuated by the replacement with fresh media (with BKA), suggesting that BKA cytotoxicity is associated with cellular metabolism. In this study, we demonstrate that BKA enhances glucose consumption rates and reduces O_2_ consumption in both 4T1 and NIH3T3 cells. It is thought that the inhibition of mitochondrial ATP production by BKA is associated with increased glycolysis for energy production in cells.

## 2 Materials and methods

### 2.1 Cell culture and assay reagents

Murine mammary carcinoma 4T1 cells were purchased from American Type Culture Collection (Manassas, VA, USA). Immortalized murine fibroblast NIH3T3 cells were purchased from RIKEN Cell Bank (Tsukuba, Japan). The cell lines were maintained in Roswell Park Memorial Institute-1640 medium (Wako Pure Chemical Industries Ltd., Osaka, Japan) supplemented with 10% heat-inactivated fetal bovine serum in a 5% CO_2_ atmosphere at 37 °C. BKA was purchased from Sigma-Aldrich Japan K.K. (Tokyo, Japan) as well kindly gifted by Prof. Y. Shinohara, Institute for Genome Research, University of Tokushima, Japan. There was no fundamental difference between the activity of the purchased and gifted BKA. The cells were seeded at 25,000 cells/well in a 96-well tissue culture plate (BM Equipment Co., Ltd., Tokyo, Japan). The following day, the media was refreshed with media containing the indicated reagents and further incubated for assay, unless otherwise indicated.

### 2.2 Measurements of glucose and lactate in media

The glucose in the media was measured using a Glucose Colorimetric/Fluorometric Assay Kit (BioVision Inc., Milpitas, CA, USA). At the indicated time, the recovered culture media were diluted 250-fold with phosphate-buffered saline (PBS), mixed with an equal volume of the reaction solution, and incubated at 37 °C for 30 min. The fluorescence (excitation wavelength/emission wavelength [Ex/Em] = 535 nm/590 nm) of the mixture was measured by a Synergy HT plate reader (Biotek, Vermont, USA), and the glucose concentration was calculated from the calibration curve. The lactate in the media was measured with a Glycolysis Cell-Based Assay kit (Cayman Chemical, Ann Arbor, MI, USA) according to the manufacturer's instructions. The collected culture media was diluted 10-fold with the assay buffer, mixed with an equal volume of the reaction solution, and incubated for 30 min at room temperature. The absorbance at 490 nm was measured on a Sunrise plate reader (Tecan Group Ltd., Männedorf, Switzerland) to calculate the lactate concentration in the media.

### 2.3 Quantification of cellular ATP

Cellular ATP was measured using a Luminescent ATP Detection Assay Kit (Abcam, Cambridge, UK). The cells were seeded at 25,000 cells/well, and the media were refreshed the following day. After 24 h of culture, the cells were washed briefly with PBS and then lysed with the lysing buffer (0.02 M sodium hydroxide). Then, 50 μL of the lysate was mixed with 25 μL of the ATP substrate buffer. The emitted luminescence was measured on a Lumat LB 9507 luminometer (Berthold Technologies GmbH & Co. KG, Bad Wildbad, Germany). Finally, 50 μL of the lysate was mixed with an equal volume of 0.02 M hydrochloric acid solution and assayed for protein using a BCA Protein Assay Kit (Pierce Biotechnology, Rockford, IL, USA). The results obtained were used to calculate the amounts of ATP from the calibration curve and normalized by the protein concentration.

### 2.4 O_2_ consumption assay

The extracellular O_2_ consumption was estimated by an increase in phosphorescence signal of an O_2_-sensing fluorophore using the Extracellular O_2_ Consumption Assay kit (Abcam plc, Cambridge, UK) according to the manufacturer's instructions. A day before the assay, 4T1 and NIH3T3 cells were seeded at 40,000 cells/well in a 96-well black clear-bottom plate and incubated in a 5% CO_2_ atmosphere at 37 °C, the media were refreshed with or without 100 μm BKA, and 10 μL of Extracellular O_2_ Consumption Reagent was added. The wells were sealed with an overlay of 100 μL mineral oil and incubated at 37 °C for 2 h. The phosphorescence signal intensity was measured by a Synergy HT plate reader (Ex/Em = 380 nm/650 nm) at 37 °C. Each result was indicated as a percentage of the untreated control.

### 2.5 JC-1 staining

To evaluate mitochondrial membrane potential (*ΔΨm*), JC-1 staining (5,5,6,6-tetrachloro-1,1,3,3-tetraethylbenzimidazolylcarbocyanine iodide; Biomol GmbH, Hamburg, Germany) was performed. JC-1 was dissolved in dimethyl sulfoxide and further diluted to 10 μM with the culture media. The diluted JC-1 was filtered and added along with 10 μM Hoechst 33342 (Dojindo Molecular Technologies, Inc., Kumamoto, Japan) to the culture. Following 60 min incubation, the tissues were washed three times with PBS and immediately analyzed on a Synergy HT plate reader for Hoechst 33342 (Ex/Em = 360 nm/460 nm) and JC-1 (Ex/Em = 530 nm/590 nm). The JC-1 results were normalized by the results of Hoechst 33342 nuclear staining and further indicated as a relative intensity compared with the result of the untreated control (30,000 cells/well, 2 mg/mL glucose).

### 2.6 Cell death assay

The cell death assay was performed using the Cytotox 96 Non-Radioactive Cytotoxicity Assay kit (Promega, WI, USA) according to the manufacturer's instructions and indicated as the percentage cell death compared with that of the lysed cells by freeze-thawing.

### 2.7 Statistical analysis

All experiments were performed at least three times, and the representative results are presented. Statistical evaluation of differences between the experimental groups was conducted using analysis of variance and two-tailed unpaired Student's t-tests. P-values < 0.05 were considered statistically significant.

## 3 Results

### 3.1 BKA enhances glycolysis

**Fig. 1:**
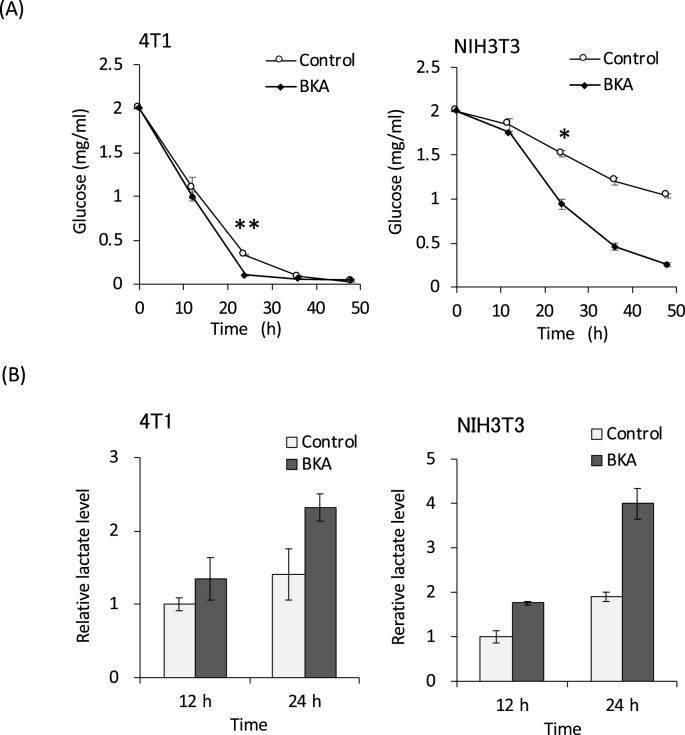
**Enhanced glucose consumption by BKA.** 4T1 and NIH3T3 cells were cultured with or without 100 μM BKA, and the culture media were sampled at the indicated time. The glucose (A) and lactate (B) concentrations in the cultured media were evaluated as described in the Materials and Methods. *p < 0.0005, **p < 0.00001.

We previously used a WST-8 reagent, which reflects the cellular NADH level, to demonstrate that BKA cytotoxicity in 4T1 cells is dependent on the cell number and BKA dose. The BKA cytotoxicity was reduced by refreshing the media 12 h after stimulation. Together with the cell number-dependent cytotoxicity, we suspected that the lack of nutrition in the media was caused by the BKA stimulation. The measurements of the glucose in the culture media indicated that BKA increased the glucose consumption rate in both 4T1 and NIH3T3 cells (Fig. 1A). As shown in Fig. 1A, the basal rate of glucose consumption is much higher in 4T1 than NIH3T3 cells. This observation is indicative of the Warburg effect, whereby cancer cells favor metabolism via glycolysis, even under aerobic conditions [12]. As a result of the increased glucose consumption caused by BKA, the glucose in the media was almost depleted in 4T1 cells. On the other hand, glucose was still present in NIH3T3 cells at 24 h, although glucose consumption was increased in the presence of BKA. To confirm the enhanced glycolysis, we measured the level of lactate in the culture media. The lactate level was increased in the presence of BKA in both 4T1 and NH3T3 cells (Fig. 1B), indicating that BKA stimulates glycolysis in both cell types.

### 3.2 BKA reduces ATP in 4T1 cells

**Fig. 2:**
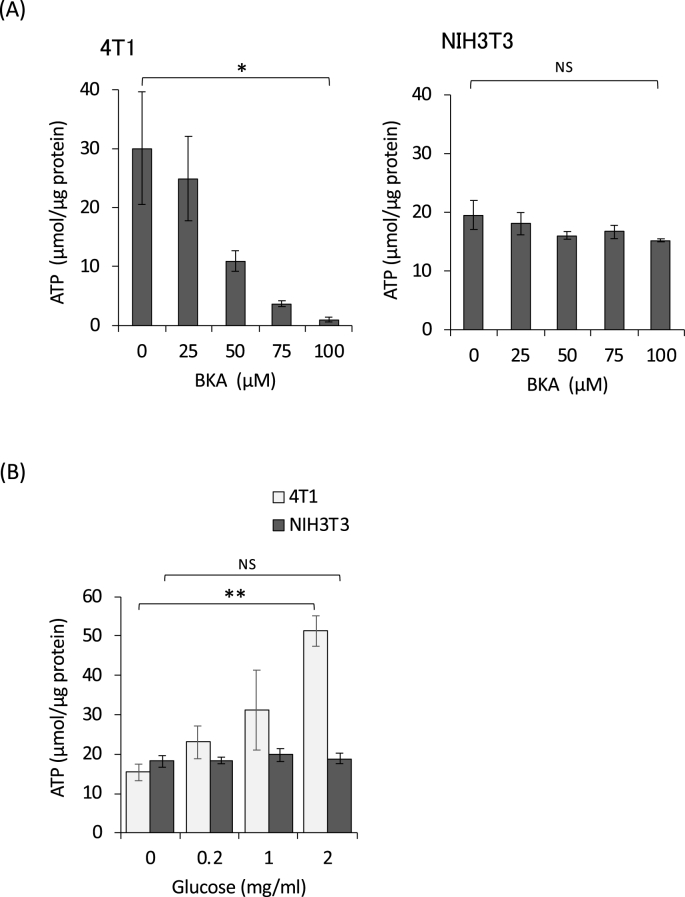
**BKA decreases cellular ATP in 4T1 cells but not NIH3T3 cells.** (A) The cells were cultured with the indicated concentration of BKA for 24 h, and the intracellular ATP was measured. (B) The cells were cultured with the indicated concentration of glucose for 24 h and the intracellular ATP was measured. *p < 0.05, **p < 0.0005, NS: not significant.

Next, we examined the level of cellular ATP in the presence of BKA. BKA reduced ATP in 4T1 cells in a dose-dependent manner but not in NIH3T3 cells (Fig. 2A). To evaluate the effect of glucose in the media on cellular ATP, we cultivated the cells in glucose-reduced media. Fig. 2B shows that the level of ATP in 4T1 cells was higher than that of NIH3T3 cells under standard conditions (2 mg/mL of glucose); however, it was decreased with the reduced concentration of glucose in the media. Interestingly, the ATP in NIH3T3 cells remained constant, even without glucose for least 24 h. For rapidly proliferating cells like cancer cells, glucose metabolism is essential for energy production and macromolecular synthesis [13]. The deprivation of glucose in the media due to the increased glucose consumption caused by BKA is probably critical for 4T1 cell viability.

### 3.3 BKA suppresses O_2_ consumption

**Fig. 3:**
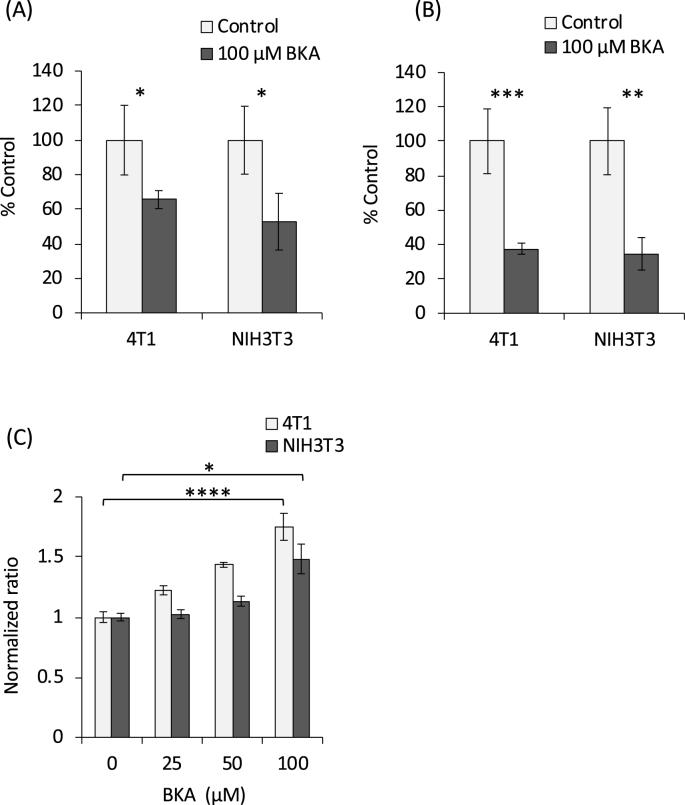
**Effect of BKA on mitochondrial respiration.** Following overnight culture, the media were refreshed with media containing BKA and the O_2_ probe as described in the Materials and Methods section. The treated cells were further incubated at 37 °C for 2 h, and the phosphorescence was measured (A). The cells were treated similarly, and then, 6 h later, the O_2_ probe was added. The cells were further incubated for 2 h and the phosphorescence was measured (B). The effect of BKA on the *ΔΨm* was evaluated by JC-1 staining as described in the Materials and Methods section (C). *p < 0.05, **p < 0.01, ***p < 0.005, ****p < 0.001.

The inhibition of ATP production by BKA has been demonstrated using isolated mitochondria [14]. Together with the above results, it was conceivable that BKA also suppresses ATP production in the cellular mitochondria. Therefore, we next evaluated mitochondrial ATP synthesis via oxidative phosphorylation (OXPHOS) by measuring the changes in the O_2_ level in the media. Unlike our initial expectations ascribed to the disadvantageous effect of BKA on the membrane permeability owing to its polar structure, the reduced O_2_ consumption was observed soon after BKA stimulation in both 4T1 and NIH3T3 cells (Fig. 3A). When the O_2_ consumption was measured 6 h after BKA stimulation, the suppression was more remarkable (Fig. 3B). To further evaluate the mitochondrial function, the mitochondrial membrane potential (*ΔΨm*) was determined by JC-1 staining (Fig. 3C). The red fluorescence, attributed to the J-aggregate of JC-1, increased in a dose-dependent manner in both 4T1 and NIH3T3 cells, suggesting *ΔΨm* elevation. Taken together, these results demonstrate that mitochondrial ATP synthesis is suppressed by BKA, while the activity of the electron transport chain and the inner mitochondrial membrane integrity remain intact.

### 3.4 BKA induces cell death in glucose-reduced media

**Fig. 4:**
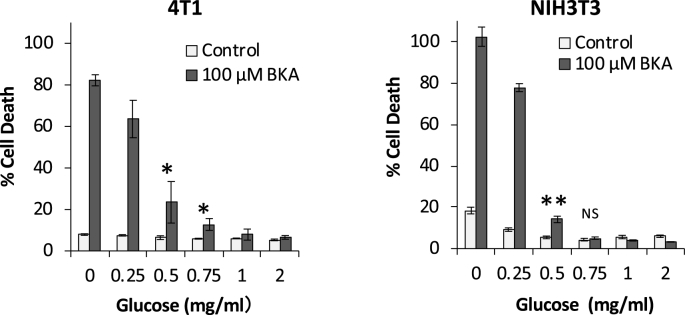
**BKA induces cell death in low glucose media.** 4T1 and NIH3T3 cells were cultured in the media with the indicated glucose concentrations for 24 h. Cell death was evaluated as described in the Materials and Methods. *p < 0.05, **p < 0.005, NS: not significant.

We next examined the effect of glucose concentration in the media on BKA cytotoxicity. Although BKA markedly reduced cellular ATP in 4T1 cells, the cells remained viable under conditions of normal glucose concentration (2 mg/mL glucose; Figs. 2A and 4). When glucose in the media was decreased to <0.75 mg/mL in 4T1 cells and <0.5 mg/mL in NIH3T3 cells, cell death began to be observed in the presence of BKA. This result demonstrates that in the presence of BKA, the source of energy for the survival of both cell types was heavily dependent on the glucose that was produced when ATP was suppressed in the mitochondria. The small difference in the threshold of glucose concentrations between 4T1 and NIH3T3 cells may reflect a difference in their dependency on glycolysis.

## 4 Discussion

The ATP synthesized in mitochondria is transported to the cytosol in exchange for cytosolic ADP, which is carried out by an ANT protein. The properties of ANT proteins are unique, in that they also form mitochondrial permeability transition pores (MPTPs) with voltage-dependent anion channel and cyclophilin D [15]. Under pro-apoptotic conditions, MPTPs are increased, and harmful proteins, such as cytochrome *c*, are released through the MPTPs to the cytosol to eventually activate caspases and cause apoptotic cell death. The role of ANT in MPTPs for mitochondria-dependent apoptosis have been described in many papers [16]. However, ANT-deficient hepatocytes undergo mitochondria-dependent apoptosis. So, the role of ANT in MTPTs is still controversial [17,18]. We previously reported that BKA is cytotoxic to 4T1 tumor cells, demonstrated by a CCK-8 kit consisting of a water-soluble formazan dye, WST-8 regents with an electron mediator, with the color development of the WST-8 reagents reflecting the cellular NADH level [19]. In fact, we observed that BKA selectively decreased the cellular NADH in tumor 4T1 cells, but not in NIH3T3 cells (data not shown), as well as the cellular ATP (Fig. 1). In this paper, we also demonstrated that the BKA increases glucose consumption in both 4T1 and NIH3T3 cells due to enhanced glycolysis. The evaluation of O_2_ in the culture media demonstrated that BKA reduces O_2_ consumption in both cell types, indicating the suppression of OXPHOS in mitochondria, which would result in enhanced glycolysis due to the lack of ATP production.

While OXPHOS was reduced, the *ΔΨm* was increased by BKA in 4T1 and NIH3T3 cells. A correlation between the bioenergetic activity of mitochondria and glucose consumption has been reported [20,21]. For example, the H^+^-ATP synthase inhibitor oligomycin reduces the O_2_ consumption rate and stimulates aerobic glycolysis. In addition, it has been reported that oligomycin enhances *ΔΨm* [22].

BKA-induced cell death in both types of cell lines under conditions of low glucose without any apoptotic signatures. The small difference in the glucose concentration threshold for cell death may be attributed to the dependency on glucose for their energy production, supposed from the difference observed in glucose consumption. The BKA-induced cell death in the low glucose environment appears to be necrosis. It is thought that the defect of energy production in both mitochondria and glycolysis results in cell death. In addition, we previously reported that BKA inhibits autophagic development [11]. Autophagy inhibition may also contribute to cellular energy deprivation. The environment of tumor tissues *in vivo* is considered to be one of low glucose due to the high glucose consumption of cancer cells and the disordered vasculature. Thus, the ANT proteins could be a target for cancer therapy to suppress ATP production in the mitochondria under glucose deprivation.

## Conflicts of interest

None declared.

## Funding

This work was supported by JSPS KAKENHI (grant numbers JP26293004, JP2667003, and JP16H01157 to M.S. and JP17K07219 to A.K.) and the Research Program for CORE lab of "Dynamic Alliance for Open Innovation Bridging Human, Environment and Materials" in "Network Joint Research Center for Materials and Devices" in Japan (Grant number 20166011 to A.K.).

## Author contributions

A.K. designed and prepared all experiments and wrote the manuscript. T.I. performed the glucose, ATP, and NAD^+^/NADH assays. M.S. contributed to the Discussion section of the paper.
